# Esophageal Plasmacytoma Revealed by Persistent Hiccups: A Case Report and Literature Review

**DOI:** 10.1155/2022/2242768

**Published:** 2022-01-31

**Authors:** Khaoula Khalil, Fatima Ezzahra Lahlimi, Illias Tazi

**Affiliations:** Department of Hematology and Bone Marrow Transplantation, Mohammed VI University Hospital, Cadi Ayyad University, Marrakesh 40080, Morocco

## Abstract

**Introduction:**

Solitary extramedullary plasmacytoma (SEP) is a rare neoplasm that is derived from monoclonal proliferation of plasma cells in the soft tissues or organs arising outside the bone marrow. It is present in about 3% of all plasma cell tumors and originates mainly from the upper respiratory tract and nasopharynx. Involvement of the esophagus is exceptionally seen in cases of SEP. *Case Presentation*. We report a novel case of a 74-year-old male patient attended with a 6-month history of hiccupping further associated with dysphasia and weight loss all caused by esophageal plasmacytoma. Histological and immunohistochemical examination of the tumor confirmed the diagnosis of plasmacytoma. Workup for the multiple myeloma came out to be negative, thus confirming the diagnosis of SEP. The patient was treated with radiotherapy alone, leading to complete remission (at 30 months of follow-up).

**Conclusion:**

Esophageal plasmacytoma, an exceptional presentation of extramedullary plasmacytoma, should be kept in mind while dealing with patients presenting with intractable hiccups.

## 1. Introduction

Solitary extramedullary plasmacytoma (SEP) is a rare form of plasma cell dyscrasia derived from monoclonal proliferation of plasma cells and located in the soft tissues or organs arising outside the bone marrow [1]. The gastrointestinal tract accounts for less than 5% of SEP [2]. The esophagus appears to be the least common location for the SEP to originate. We report a novel case of esophageal plasmacytoma in a 74-year-old male patient disclosed by refractory hiccupping and treated with radiation monotherapy alone. A brief overview of the relevant literature is given focusing on the presentation, the diagnosis, and the treatment of an exceptional site and presentation of SEP.

## 2. Case Presentation

**Figure 1:**
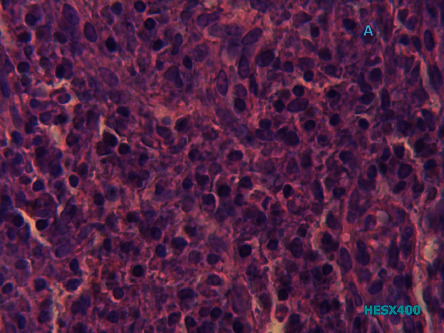
Histological examination of esophagus biopsy showing the presence of sheets of plasma cells.

**Figure 2:**
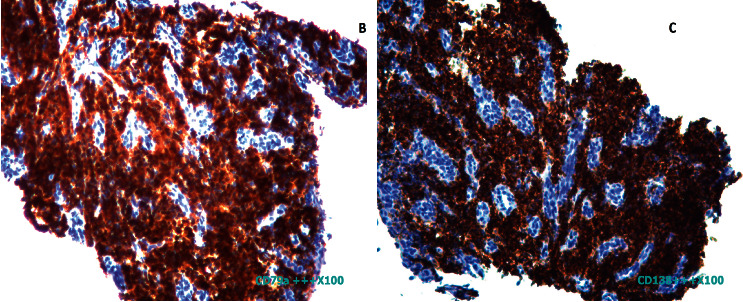
(a) Immunohistochemical examination of biopsy sample showing positivity in majority of tumor cells for CD79a and (b) CD138.

A 74-year-old man presented, in October 2018, with a 6-month history of persistent hiccupping. Since 3 months before the visit, his hiccups had worsened disturbing his sleep and daily life and were associated with intermittent dysphagia and recurrent postprandial vomiting. There was no history of alcohol consumption or cigarette smoking. He did not present with fever, night sweats, hiatus hernia symptoms, abdominal pain, a recent change in diet, bone pain, or any pathological fracture in the recent past. Physical examination revealed an old man in no apparent distress except for hiccups. At presentation, his temperature was 37.3°, pulse rate was 82/min, respiratory rate was 18/min, and blood pressure was 120/70 mmHg. He did not present any significant alterations beyond weight loss. There was no pallor, icterus, lymphadenopathy, bone pains, or any bleeding manifestations such as ecchymosis or petechiae. Esophagoscopy identified a 4-centimetre circumferential ulcerated mass of the distal third of the esophagus. The biopsy results of the esophagus showed abundant sheets of small lymphoid mononuclear cells (plasma cells) (Figure 1). Immunohistochemistry analysis showed positive expression for CD138, CD79a, and EMA and negative expression for CD20, CD3, and pan-cytokeratin. Ki-67 index value was 20% (Figure 2). These pathological features were consistent with extramedullary plasmacytoma diagnosis.

**Figure 3:**
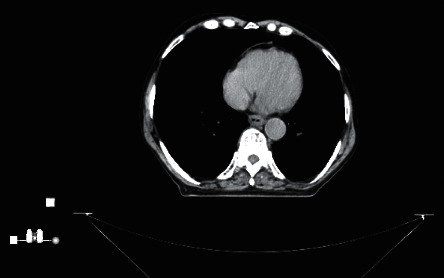
Pretreatment computed tomography showing circumferential esophageal wall thickening located at the distal third of the esophagus.

Computed tomography of the chest and abdomen revealed a circumferential esophageal wall thickening of 9 mm that was located at the distal third of the esophagus measuring 38.3 mm (Figure 3). No enlarged lymph nodes, other organ involvement, or osteolytic lesions were found. Complete blood count (CBC) showed hemoglobin of 13.5 g/dl, total leucocyte count of 6400/mm^3^, normal platelet count of 211,000/mm^3^, and erythrocyte sedimentation rate (ESR) of 12 mm/1st hour and B2 microglobulin of 3.13 mg/L. Serum creatinine and calcium levels were within the normal range.

Bone marrow tests (aspiration and biopsy) did not show the presence of myeloma cells. Serum protein electrophoresis and immunofixation were also negative for any monoclonal gammopathy.

Urine examination was negative for Bence-Jones proteins. All the investigations listed above confirmed the diagnosis of solitary extramedullary plasmacytoma of the esophagus. The patient was referred to the radiotherapy department where three-dimensional conformal radiotherapy (3DCRT) was applied to the involved site (oesophagus) with curative intent. The prescribed dose of 40 Gy was delivered in 20 fractions over a period of 4 weeks. Radiotherapy was well-tolerated, and the patient's hiccupping resolved progressively during treatment with only a few episodes noted while eating or drinking that have persisted after the end of treatment. During the last thirty months of follow-up, no recurrence or progression to myeloma were noted.

## 3. Discussion

Solitary extramedullary plasmacytoma (SEP) is a proliferation of neoplastic monoclonal plasma cells in soft tissues outside the bone marrow. It accounts for 3% of plasma cell tumors and involves males in 75% of cases [1, 2]. The diagnosis of SEP is based on the exclusion of systemic involvement and an absence of end-organ damage attributable to plasmacytoma [3]. It arises mainly from the upper respiratory tract (nasal cavity, paranasal sinuses, and nasopharynx). The stomach and small bowel are the most usual primary sites when the gastrointestinal system is involved. The esophagus is an extremely rare primary location for the SEP to originate, and only 6 cases of esophageal extramedullary plasmacytoma have been reported in the literature to date. Morris and Pead described a case of a 59-year-old woman who presented with weight loss and progressively worsening intermittent dysphagia [4]. The SEP was a protuberant mass that occurred in the lower esophagus. Ahmed et al. reported a case of a 67-year-old man who had dysphagia, weakness, and weight loss [5]. The case of Davies and Boxer occurred in a 69-year-old man who only had a 2-week history of dysphagia with no other significant alterations [6]. The tumor was polypoid, measured 4 cm in diameter, and involved the distal one-third of the esophagus. Chetty et al. reported a case of a 58-year-old man who presented with dysphagia for solids over a period of 2 months [7]. The fifth case belongs to Rimmer et al. describing a 62-year-old man, with no dysphagia, who presented with cardiac chest pain and was worked up for possible acute myocardial injury [8]. Zhou et al. reported in 2012 the sixth case of an 80-year-old-woman with a primary isolated extramedullary plasmacytoma on the left posterior wall of the proximal esophagus that was treated with endoscopic submucosal dissection [9].

Plasmacytomas commonly show a protuberant mass on endoscopy. The most common clinical presenting features of esophageal plasmacytoma are similar to those of carcinomas such as worsening dysphagia due to mass effect, anorexia, and weight loss [5]. Hiccupping, the primary and main ongoing symptom in our case, has never been reported in the literature. It may be due to a direct effect of the tumor itself or to a secondary dilatation of the esophagus above a stenotic lesion leading to raised pressure that stimulates a reflex action or perhaps to the disruption to esophageal motility that occurs in esophageal cancer [10].

Despite the consideration of local radiotherapy as the preferred treatment modality for solitary plasmacytoma, there are no treatment guidelines for esophageal plasmacytoma. Many authors reported achieving complete remission with radiotherapy followed by esophagectomy: the gold standard treatment for esophageal cancer [11]. We opted for radiotherapy alone using 3-dimensional conformal radiotherapy (3DCRT) with curative intent in a daily fraction of 2 Gy, 5 days a week. The dose used for our patient was 40 Gy as recommended by the International Lymphoma Radiation Oncology Group. Interestingly, Elsayad et al. have shown in their international multicenter analysis that using radiotherapy dose-escalation (above 40 Gy) and modern irradiation techniques as intensity-modulated radiotherapy (IMRT) and proton therapy seem to improve the local control and reduce the rate of relapse in patients with solitary bone or solitary extramedullary plasmacytoma, without a significant effect on survival rates [12]. Imaging examinations, such as positron emission tomography (PET) computed tomography (CT), are required for the management of suspected SEP, as well as to monitor the therapeutic response to radiotherapy and the possibility of recurrence and progression of into multiple myeloma [13].

A follow-up CT scan 6 months after 3DCRT showed a normal structure of the esophagus. Endoscopic examination with multiple biopsies of the plasmacytoma site and surrounding tissues attested to the good radiotherapy results showing normal esophageal mucosa without plasma cell infiltration of the scars and the surrounding tissue. Also, neither recurrence nor progression to myeloma was noted during the last thirty months of follow-up.

## 4. Conclusion

We have presented a novel case of an extramedullary plasmacytoma revealed by intractable hiccupping and effectively treated with local radiotherapy. Hiccups are common and often idiopathic, but persistent hiccups could be an unrecognized symptom of many esophageal cancers not only common ones like carcinoma but also the rare ones like extramedullary plasmacytoma.
